# Revisiting Antibiotic-Impregnated Cement Spacer for Diabetic Osteomyelitis of the Foot

**DOI:** 10.3390/antibiotics13121153

**Published:** 2024-12-01

**Authors:** Farouk Khury, Ihab Karkabi, Elias Mazzawi, Doron Norman, Eyal A. Melamed, Eli Peled

**Affiliations:** Division of Orthopedic Surgery, Rambam Health Care Campus, Haifa 3109601, Israel

**Keywords:** diabetic foot, resection arthroplasty, antibiotic cement-spacer, osteomyelitis, antibiotic PMMA

## Abstract

Introduction: Despite the rising global awareness and improvement of socioeconomic and living standards, the prevalence of diabetic osteomyelitis (DOM) and its complications has been increasing rapidly. This study aims to investigate the long-term prognosis of DOM of the foot treated using antibiotic-impregnated cement spacer (ACS) and the contributing risk factors for reoperation. Methods and Materials: We retrospectively reviewed the data of 55 diabetic patients with Meggitt-Wagner Grade IIB wounds diagnosed with osteomyelitis of the foot, treated in our institution with excessive debridement, excision of the infected tissue, and implantation of antibiotic-impregnated cement spacer fixed with a Kirschner wire. Descriptive statistics, including patient demographics, were analyzed. Statistical analysis was performed using point-biserial correlation and a Chi-square test with Cramer’s V effect-size estimation to determine the relationship between reoperation and various parameters. Results: 55 patients (36 (65.45%) males and 19 (34.55%) females) with a median age of 64 (39–84) years were thoroughly analyzed throughout a median follow-up of 884 days (2–4671 days). Of the entire cohort, 29 (52.72%) patients achieved primary successful infection eradication without any further intervention, and 8 (14.54%) patients were successfully treated using a secondary procedure. More than half of the reoperated patients underwent the secondary intervention within less than a month after the primary ACS. When assessing correlation, age (r = 0.28, *p* = 0.04), gender (r = 0.31, *p* = 0.02), *Staphylococcus aureus* (r = −0.10, *p* = 0.04), and the use of gentamicin-only antibiotic cement spacer (r = 0.34, *p* = 0.01) demonstrated statistically significant correlation to reoperation. 89.18% of the patients who achieved infection eradication did not undergo cement removal. Conclusions: ACS has shown excellent results in eradicating bone infection with up to 7.23 years of follow-up, acting as a structural stabilizer, preventing soft tissue contractures, and delivering highly concentrated local antibiotic treatment both to soft tissue and bone. Regardless, specific factors should be thoroughly evaluated prior to surgery, as advancing age, gender, and the use of gentamicin-only antibiotics appear to be positively associated with a higher likelihood of reoperation. Conversely, infections caused by cultured *Staphylococcus aureus* seem to be inversely related to reoperation.

## 1. Introduction

Despite the rising global awareness, improvement of socioeconomic and living standards, and development of wound treatment strategies [1,2], the prevalence of diabetes and its complications has been increasing rapidly [3]. One of the most devastating outcomes of an untreated diabetic foot ulcer, most often related to advanced peripheral neuropathy, is osteomyelitis (DOM), which has been associated with significant morbidity, prolonged hospitalizations, a higher risk for amputation, and a growing financial burden [4,5]. DOM is often coupled with peripheral vascular disease, insufficient adherence to foot care guidelines, and foot structural abnormalities [6,7].

Preclinical research has provided valuable insights into the pathophysiology and potential therapeutic targets for diabetic osteomyelitis. Studies using animal models have demonstrated the efficacy of various antimicrobial agents and the role of immune response modulation in managing bone infections [8,9]. These findings have paved the way for developing advanced clinical strategies aimed at improving patient outcomes.

The clinical gold standard for treating DOM involves a combination of surgical debridement and targeted antibiotic therapy. Furthermore, the successful treatment relies on a multidisciplinary team approach to eradicate the infection [10]. From the surgical standpoint, the traditional treatment consisted of extensive debridement and selective excision of the infected tissue [11]. However, standard debridement techniques can be challenging and may not always be effective in eradicating the infection, especially in cases of deep or widespread osteomyelitis. In recent years, the concept of excising infected tissue while preserving the external structures [12] has been increasing in popularity. Although this concept, in theory, minimizes structural damage and enhances cosmetic appearance by avoiding amputation, it could potentially compromise the structural integrity and perpetuate the infection within the unoccupied space.

First described in the early 1980s, antibiotic-impregnated cement spacer (ACS) has been proposed as a solution for the aforementioned issues [13,14,15]. Acting as a structural stabilizer, preventing soft tissue contractures, and delivering highly concentrated local antibiotic treatment both to adjacent soft tissue and bone, ACS has shown successful results in eradicating bone infection, with published success rates up to 91.3% [16,17,18,19,20,21,22,23].

Despite the previous publications [16,17,18,19,20,21,22,23], to the best of our knowledge, no study has yet investigated the long-term prognosis of DOM of the foot treated using ACS and the contributing risk factors for reoperation. Therefore, we conducted this retrospective study with a long follow-up time period in order to answer the following questions: First, what are the primary outcomes of DOM treated with ACS? Second, is there a correlation between reoperation and patients’ characteristics (gender, age, and comorbidities)? Third, is there a correlation between reoperation and the cultured bacterial organism? Fourth, is there a correlation between reoperation and preoperative serum inflammatory marker values? Fifth, is there a correlation between reoperation and the type of antibiotic-impregnated cement spacer used?

## 2. Materials and Methods

### 2.1. Study Design

This study is a retrospective review with chart data analyses of patients with diabetic osteomyelitis of the foot treated with excision of the infected tissue and implantation of antibiotic-impregnated cement spacer at one tertiary referral center.

Following approval from our institutional review board, we searched our database, which primarily yielded 82 potential diabetic patients with Meggitt-Wagner Grade IIB [24] wounds, clinically or radiologically diagnosed osteomyelitis of either the ray, proximal, middle, or distal phalanx, diabetic peripheral neuropathy, and adequate vascular perfusion, who underwent ACS in our institution. Among those, 5 and 22 patients had necrotizing fasciitis and were lost to follow-up, respectively. We were left with 55 patients treated with ACS for severe infection with osteomyelitis without signs of gangrene. Sample size analysis conducted using the G*Power program (version 3.1.9.7, Heinrich-Heine-Universität Düsseldorf, Düsseldorf, Germany) with a type I error rate of alpha 0.05, a power of 85%, and an effect size of 0.3 yielded a total sample of 52 patients.

### 2.2. Surgical Technique

All procedures were performed under ankle block anesthesia with 2% lidocaine and either sedation or general anesthesia. The surgery included excision and debridement of the necrotic and infected soft tissue and bone until reaching macroscopically unremarkable tissue. Tissue samples were sent to the pathology and microbiology labs. Following adequate irrigation and drying, a polymethylmethacrylate (PMMA) premixed with gentamicin (Palacos R + G, Heraeus Medical GmbH, Wehrheim, Germany) was mixed with an antibiotic powder (either vancomycin or amikacin or a combination of both). During its plastic phase, the antibiotic-mixed PMMA, termed cement spacer, was inserted into the debrided void and stabilized with Kirschner wires under intraoperative fluoroscopy. Excess cement was removed, and the wound was irrigated with saline solution after the hardening of the cement. The wound was sutured using nylon stitches. Postoperatively, patients were treated with antibiotics and were hospitalized until clinical and serological signs of infection eradication were seen. The stitches were removed in the first outpatient follow-up visit, 21 days postoperatively. We continued to follow the patients once a month for three successive visits and then every 3 months on a routine basis. The outcomes were evaluated based on the continued presence of infection and whether the ACS appeared to provide adequate stability.

### 2.3. Data Analyses

Patient demographics, including age, gender, and comorbidities (diabetes mellitus type 1 or 2, arterial hypertension, peripheral vascular disease, ischemic heart disease, chronic obstructive pulmonary disease), type of cultured organisms, type of antibiotics used, serum inflammatory marker values (C-reactive protein (CRP) and white blood cell count (WBC)), dates of hospitalization, operations, and discharge, and type of antibiotics mixed with the PMMA throughout the whole inpatient treatment, were recorded. Descriptive statistics, including patient demographics, were analyzed. The statistical model consisted of two tests: point-biserial correlation to determine the relationship between reoperation and the continuous values (age, CRP, and WBC) and a Chi-square test of independence to assess the correlation between reoperation and the categorical parameters (gender, comorbidities, type of cultured bacterium, and type of antibiotic cement used) with effect-size estimation using Cramer’s V. A *p*-value of <0.05 was considered to be statistically significant. All statistical analyses were performed using IBM SPSS software version 23 (IBM Corporation, Armonk, NY, USA).

## 3. Results

The data on 55 patients (36 (65.45%) males and 19 (34.55%) females) with a median age of 64 (39–84) years were thoroughly analyzed throughout a median follow-up of 884 days (2–4671 days). Of the total number of patients, 51 (92.72%) and 4 (7.27%) patients had diabetes mellitus type 1 and 2, respectively; 44 (80%) patients had arterial hypertension, 15 (27.27%) patients had peripheral vascular disease, and 21 (38.18%) patients had ischemic heart disease. None of the patients were previously diagnosed with chronic obstructive pulmonary disease.

### 3.1. Outcomes

**Figure 1:**
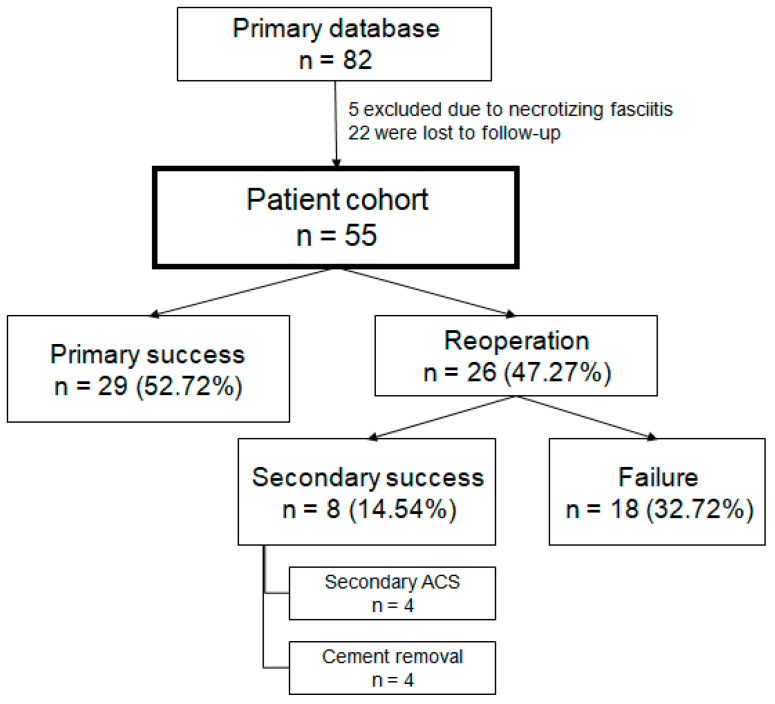
Flow diagram presenting patient cohort with preliminary outcomes. n, number of patients; ACS, debridement with implantation of antibiotic-impregnated cement-spacer.

Of the entire cohort, 29 (52.72%) patients achieved primary successful infection eradication without requiring any further intervention, with up to 8.75 years of follow-up. Of these 26 (19 males and 7 females) (47.27%) patients required reoperation, 8 (14.54%) of which were successfully treated using either a secondary debridement and ACS or cement removal with up to 3.97 years of follow-up, and 18 (32.72%) of which failed the primary ACS and required excessive debridement and amputation of the infected bone and soft tissue (Figure 1). Out of the entire cohort, 37 (67.72%) patients achieved absolute infection eradication following either primary or secondary intervention with up to 7.23 years of follow-up. The cement spacer was not removed in 89.18% of the successful cases.

**Figure 2:**
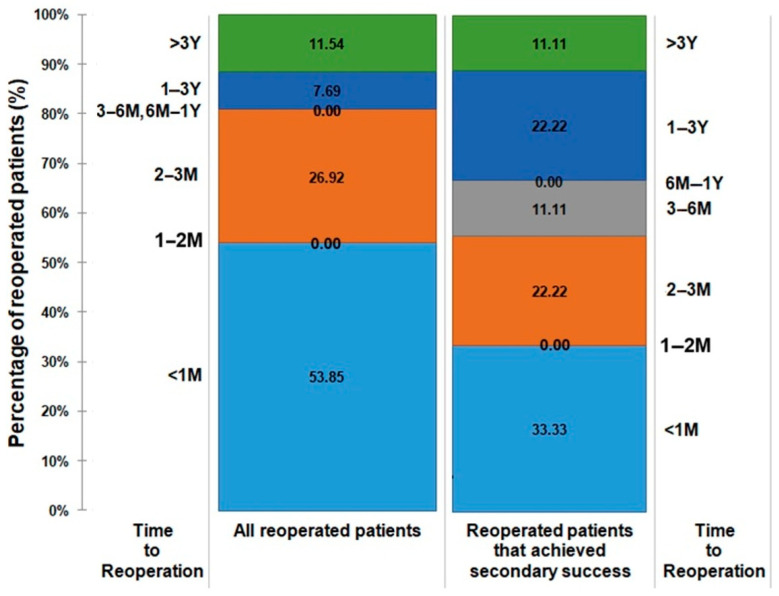
Bar chart demonstrating the percentage of all reoperated patients and the reoperated patients who achieved secondary eradication success in relation to the time frame in which they were operated. M, month; Y, year; <1 M, less than one month; 1–2 M, between one and two months; 2–3 M, between two and three months; 3–6 M, between three and six months; 6 M–1 Y, between six months and one year; 1–3 Y, between one and three years; >3 M, more than three years.

The majority (53.85%) of all reoperated patients underwent a secondary intervention within less than a month, 26.92% within two to three months, and 7.69% within one to three years, and 11.54% after three years. A similar trend was observed in patients who achieved infection eradication following secondary operation (Figure 2). In the time period of six months to a year following the primary ACS, no patients required a reoperation.

### 3.2. Correlation Between Reoperation and Patients’ Characteristics

When assessing the relationship between patients’ characteristics and a secondary intervention, age and gender were the only variables to exhibit statistically significant (*p* = 0.04 and 0.02, respectively) results, with positive correlations of 0.28 and 0.31, respectively. Patients’ comorbidities did not reveal any significant correlations (Table 1).

### 3.3. Correlation Between Reoperation and Preoperative Cultured Bacterial Organism

Out of all analyzed organisms, *Staphylococcus aureus* was the only bacterium to demonstrate statistical significance (*p* = 0.04), with a rather negative correlation of −0.1 (Table 2).

### 3.4. Correlation Between Reoperation and Serum Inflammatory Markers

CRP and WBC did not reveal any statistically significant correlation values (Table 3).

### 3.5. Correlation Between Reoperation and Type of Antibiotic-Impregnated Cement Spacer Used

Patients who underwent implantation of gentamicin-only cement spacer demonstrated a statistically significant (*p* = 0.01) positive correlation (r = 0.34) to further intervention. The addition of vancomycin or amikacin to the premixed gentamicin-containing cement spacer did not significantly correlate to reoperation rate (Table 4).

## 4. Discussion

This retrospective analysis of 55 patients diagnosed with DOM of the foot has demonstrated that sufficient debridement and local antibiotic release using cement spacer can efficiently reduce the rate of further procedures and reduce healing time, as more than two-thirds (67.72%) of the entire cohort achieved absolute infection eradication following either primary or secondary intervention with up to 7.23 years of follow-up. This study is, to the best of our knowledge, the first of its kind to investigate the long-term outcomes of such a large patient cohort throughout more than 12 years of follow-up.

ACS has proven itself to be a successful alternative for salvage of DOM in select cases where radical amputation would generally be indicated, with a 52.72% primary infection eradication rate (Figure 1). Even though this observation has been previously noted [16,17,18,19,20,21,22,23], earlier studies [16,17,19,20,21,22,23] reported a rather short follow-up time, with the longest being 6.3 years by Elmarsafi et al. [18]. A longer follow-up is clinically relevant since 11.11% of our investigated cohort required a secondary ACS after 3 years (Figure 2) of being infection-free; hence previous publications that did not sufficiently follow up on their patients might have missed infection recurrence.

In contrast to previous studies [16,18,19,20,21,22,23], the overwhelming majority (89.18%) of the patients who achieved infection eradication did not undergo cement removal. This finding suggests that ACS, in its role as a permanent spacer, might act as a structural stabilizer both for bone and soft tissue, producing a thick biofilm that becomes an induced membrane capable of secreting cytokines needed for tissue healing [16,25]. Particularly in cases of metatarsal head resection, the cement spacer can act as a substitute and can participate in weight-bearing, avoiding a flail distal phalanx prone to infection. Furthermore, local stability can minimize uneven load distribution, transfer lesion, under adjacent joints, and avoid contractures [26,27]. Although it has been theorized that in cases of incomplete wound healing, bacterial colonization of the cement spacer might be possible, and so cement removal following infection resolution is advised [23,28,29,30], this finding is irrelevant to our study as there were no documented cases of incomplete wound healing. Moreover, in our institution we do not recommend performing ACS in cases where primary skin closure cannot be achieved.

Patients’ characteristics and comorbidities can help strategize the treatment and identify potential candidates for a successful ACS. Using statistical analysis, we found that out of all investigated patients’ parameters, age and gender were the only variables that exhibited statistically significant positive correlation (r = 0.28 and 0.31, *p* = 0.04 and 0.02, respectively) to reoperation (Table 1). This finding hints that older patients might not primarily benefit from ACS. Causes of this observation can be the advanced stage of peripheral neuropathy, weakened immune response, and reduced blood flow [31]. Interestingly enough, peripheral vascular disease did not statistically correlate to reoperation rate. This might be due to the low frequency (27.27%) of patients in our cohort who had this morbidity. Nonetheless, future studies are needed to further investigate this issue as well as to find a recommended cut-off age for ACS. Although preliminary results demonstrated that more males required a secondary intervention than females (*n* = 19 vs. 7 patients), and that gender has a positive significant correlation to reoperation, it should be emphasized that 65.45% of the investigated cohort are male subjects, which might be an important confounding factor.

Diabetic foot ulcers that progress to DOM often harbor multiple bacteria, with *Staphylococcus aureus* being the predominant one [28,32]. In our study, cultured *Staphylococcus aureus* was the only bacterium to exhibit a statistically significant (*p* = 0.04) correlation with reoperation rate. The relationship was a negative correlation (r = −0.1, Table 2), meaning that patients treated with ACS for *Staphylococcus aureus* infection were less likely to undergo further interventions. Even though this finding supports the treatment using ACS, it might be clinically irrelevant in cases of emergency surgery and in cases where there is a long time interval between specimen culture and pathogen identification.

Although numerous studies [33,34,35,36,37] have been published investigating the diagnostic accuracy of serum inflammatory markers in monitoring infection persistence, the findings in the literature are somewhat inconsistent. While most of the publications reported declining marker values following treatment initiation, the specificity and sensitivity, particularly of CRP and WBC for diagnosing DOM, were less than 0.9 [35]. Furthermore, specific cut-off values could not be determined [34]. In our analysis, no statistically significant correlation was noted between CRP and WBC and the reoperation status of the patient (Table 3). This finding further highlights the redundancy of relying on marker values when assessing infection eradication. Therefore, alternative methods or a combination of approaches might be necessary for definitive diagnosis.

Other than the mechanical advantages of having a cement spacer fill a void in order to increase stability, the addition of a thermoresistant antibiotic to the PMMA has been documented [16,18,22,23] to deliver high-concentration and long-duration local treatment, bypassing harmful systemic side effects. While gentamicin has historically been the first and most common antibiotic to describe the concept of local delivery [38], concern for increasing resistance has led to the use of other types of antibiotics such as vancomycin and tobramycin, which were first introduced in 2000 [39]. Furthermore, previous research [40] has shown that particularly in cases of polymicrobial infections, a combination of different classes of antibiotics might increase the elution. Similar observations were noted in our study, as the use of gentamicin-only cement spacer significantly positively correlated to reoperation rate, whereas in contrast, the addition of vancomycin contributed to a rather negative correlation (Table 4). Even though the addition of vancomycin did not demonstrate statistical significance, the synergistic action of the glycopeptide and aminoglycoside can cover both Gram-negative and Gram-positive bacteria and has longer bactericidal activity than single antibiotic-loaded bone cement [41,42]. As reported in a previous publication from our institution [23], we favor the addition of vancomycin to the gentamicin-containing cement-spacer, regardless of patients’ prior antibiotic treatment and culture results, largely due to the high probability of insensitive and unspecific culture results and unsuccessful intravenous antibiotic treatment. Nonetheless, future studies are needed to investigate the specific formulations and adjusted antibiotic dosages, especially when considering multi-agent treatment, in order to reach the optimal compromise between the antibacterial and mechanical properties of the bone cement.

Despite the interesting findings, this study has several limitations. First, it’s limited by its retrospective nature; data regarding possible confounding factors, such as various antibiotic treatments before, during, and after hospitalization, were not captured. Furthermore, due to the study’s retrospective model, no control group was investigated, which can significantly impact the study’s power. Future studies should research the outcomes of ACS in the study versus control group. Moreover, the clinical outcomes, the morbidity and mortality rate and cost-effectiveness of ACS should be investigated. Second, although, to the best of our knowledge, this study has the longest follow-up time and largest patient cohort in current literature, the small sample size might affect the outcomes. Third, the patients investigated were not stratified according to their comorbidities, age, body mass index (BMI), American Society of Anesthesiologists (ASA) score, and operating surgeon. This limitation is of clinical importance, as we have observed [23] that this operation is associated with a learning curve and that surgical expertise can lead to better outcomes.

In conclusion, the ACS technique has exhibited excellent results in terms of infection eradication, with more than two-thirds of the entire patient cohort achieving infection-free status with up to 7.23 years of follow-up. Nonetheless, certain parameters should be carefully inspected before surgical treatment, as patients’ increasing age, gender, and the use of gentamicin-only antibiotics seem to positively correlate to reoperation. Furthermore, the overwhelming majority (89.18%) of the patients who achieved infection eradication did not undergo cement removal, which is in contrast to previous studies [16,18,19,20,21,22,23], and suggests that the permanent cement spacer has a secondary role as a structural stabilizer [16,25], other than delivering local antibiotic treatment.

## Figures and Tables

**Table 1:** Correlation between reoperation and patient characteristics (age, gender, and comorbidities). *, statistically significant (*p* < 0.05).

	Correlation/Cramer’s V	Significance (*p*-Value)
Age	0.28	0.04 *
Gender	0.31	0.02 *
Diabetes mellitus type 1	0.21	0.11
Diabetes mellitus type 2	0.15	0.19
Arterial hypertension	0.09	0.48
Peripheral vascular disease	0.30	0.06
Ischemic heart disease	0.02	0.83

**Table 2:** Correlation between reoperation and preoperative cultured bacterial organism.

	Correlation/Cramer’s V	Significance (*p*-Value)
Coagulase-negative staphylococci	−0.06	0.62
*Staphylococcus aureus*	−0.10	0.04
*Corynebacterium* species	−0.10	0.44
*Streptococcus* species	0.18	0.18
*Escherichia coli*	0.036	0.79
*Klebsiella pneumonia*	0.05	0.68
*Proteus* species	0.07	0.55
*Pseudomonas aeruginosa*	0.11	0.38
*Enterococcus* species	0.22	0.09
*Streptococcus pyogenes*	0.25	0.06
*Streptococcus agalactiae*	0.25	0.06

**Table 3:** Correlation between reoperation and serum inflammatory marker values.

	Correlation	Significance
CRP	−0.03	0.81
WBC	0.23	0.10

**Table 4:** Pearson’s correlation coefficient for reoperation and type of antibiotic-impregnated cement spacer used.

	Correlation	Significance
Gentamicin	0.34	0.01
Gentamicin with Vancomycin	−0.06	0.67
Gentamicin with Amikacin	0.11	0.38

## Data Availability

The data that support the findings of this study are not publicly available.

## Abbreviations

ACS	Antibiotic-impregnated cement-spacer
CRP	C-reactive protein
DOM	Diabetic osteomyelitis
PMMA	Polymethylmethacrylate
WBC	White blood cell count
BMI	Body mass index
ASA	American Society of Anesthesiologists
